# Professor Stolz on Polypi of the Rectum in Children

**Published:** 1842-04-30

**Authors:** 


### ANALECTA.

*Polypi of the Rectum in Children.*—Professor Stolz has published in the Gazette Médicale of Strasbourg, a pamphlet on polypi of the rectum in children. This disease, he says, occurs from time to time, and has almost never been taken notice of by any of those authors who have written on diseases of children, having been taken for a prolapsus of the gut. The case which first occurred to him was in a boy five years old, and presented the following symptoms:—For eighteen months he had had frequent desire to go to stool; and for a year, at each time that he went, he had passed a red and bloody tumour, which in about five or ten minutes returned of itself. His parents, and several medical men who were consulted, believed that he laboured under prolapsus of the rectum. Professor Stolz at first was of the same opinion, and various injections were accordingly ordered. After several weeks, upon examining him minutely, he discovered that it was not a prolapsus, but a tumour of the size of a small nut, and covered with a bloody mucus, which was protruded. It was attached not very high up the gut, by a pedicle of about the thickness of two millimetres to the mucous membrane of the rectum. A ligature of silk was accordingly put round it and tied. In three days it came away; no bad symptoms followed, and the child, who had been in bad health previously, from loss of blood, soon recovered his strength. Two other cases have occurred to the professor since, and he has heard from his colleagues of several more. In one of the cases which he had, he removed the tumour by means of scissors. No blood followed at the time; but in about two hours after there was a copious hemorrhage, which put the patient's life in danger. The bleeding was arrested by compresses dipped in cold water, and by cold injections. The child made a good recovery, and soon regained his strength.

This last case is another example of the danger of making an incision in the rectum, or even in its neighbourhood, without carefully plugging the wound afterwards.—*Edinburgh Monthly Journ.* Feb. 19th, 1842.

